# Total Cerebral Small Vessel Disease Score and Cerebral Bleeding Risk in Patients With Acute Stroke Treated With Intravenous Thrombolysis

**DOI:** 10.3389/fnagi.2022.790262

**Published:** 2022-04-11

**Authors:** Houwei Du, Sangru Wu, Hanhan Lei, Gareth Ambler, David J. Werring, Hangfeng Li, Yangui Chen, Dongping Chen, Qilin Yuan, Shuangfang Fang, Ronghua Chen, Yixian Zhang, Jin Wei, Guangliang Chen, Jianhua Chen, Nan Liu

**Affiliations:** ^1^Department of Neurology, Stroke Research Center, Fujian Medical University Union Hospital, Fuzhou, China; ^2^Institute of Clinical Neurology, Fujian Medical University, Fuzhou, China; ^3^Department of Neurology, Fujian Provincial Governmental Hospital, Fuzhou, China; ^4^Department of Statistical Science, University College London, London, United Kingdom; ^5^Department of Brain Repair and Rehabilitation, University College London Queen Square Institute of Neurology, London, United Kingdom; ^6^Department of Neurology, Longyan First Hospital of Fujian Medical University, Longyan, China; ^7^Department of Rehabilitation, Fujian Medical University Union Hospital, Fuzhou, China; ^8^Department of Radiology, Fujian Medical University Union Hospital, Fuzhou, China

**Keywords:** cerebral small vessel disease, ischemic stroke, thrombolysis, cerebral bleeding risk, intracerebral hemorrhage

## Abstract

**Objective:**

The aim of this study was to investigate the association of total cerebral small vessel disease (cSVD) score with the risk of intracerebral hemorrhage (ICH) in patients with acute ischemic stroke who received intravenous thrombolysis (IVT) using recombinant tissue-plasminogen activator (rt-PA).

**Methods:**

We retrospectively reviewed clinical data from two stroke registries of patients with acute ischemic stroke treated with IVT. We assessed the baseline magnetic resonance (MR) visible cSVD markers and total cSVD score (ranging from 0 to 4) between patients with and without ICH after IVT. Logistic regression analysis was used to determine the association of total cSVD score with the risk of ICH after IVT, adjusted for cofounders selected by least absolute shrinkage and selection operator (LASSO). We additionally performed an *E*-value analysis to fully explain away a specific exposure-outcome association. Receiver operating characteristic (ROC) curve analysis was used to quantify the predictive potential of the total cSVD score for any ICH after IVT.

**Results:**

Among 271 eligible patients, 55 (20.3%) patients experienced any ICH, 16 (5.9%) patients experienced a symptomatic ICH (sICH), and 5 (1.85%) patients had remote intracranial parenchymal hemorrhage (rPH). Logistic regression analysis showed that the risk of any ICH increased with increasing cSVD score [per unit increase, adjusted odds ratio (OR) 2.03, 95% CI 1.22–3.41, *P* = 0.007]. Sensitivity analyses using *E*-value revealed that it would need moderately robust unobserved confounding to render the exposure-outcome (cSVD-any ICH) association null. ROC analysis showed that compared with the National Institutes of Health Stroke Scale (NIHSS) score alone, a combination of cSVD and NIHSS score had a larger area under the curve for any ICH (0.811, 95% CI 0.756–0.866 vs. 0.784, 95% CI 0.723–0.846, *P* = 0.0004).

**Conclusion:**

The total cSVD score is associated with an increased risk of any ICH after IVT and improves prediction for any ICH compared with NIHSS alone.

## Introduction

Current stroke guidelines recommend intravenous thrombolysis (IVT) using recombinant tissue-type plasminogen activator (rt-PA) as the first-line treatment for acute ischemic stroke (Chinese Society of Neurology and Chinese Stroke Society, 2018; Powers et al., 2018; Berge et al., 2021). However, approximately 7.0–27.0% of patients treated with IVT using rt-PA experienced intracerebral hemorrhage (ICH) (Hacke et al., 2008; Dorňák et al., 2018; Anderson et al., 2019), which might be related to unfavorable clinical outcomes. Previous efforts have been made to identify several risk factors for developing ICH after IVT, including aging, hypertension, atrial fibrillation, and stroke severity (Mazya et al., 2012; Whiteley et al., 2012). Cerebral small vessel disease (cSVD) refers to a group of age-related (but not clearly age-derived) pathological processes with various etiologies that affect the microvasculature in the brain and might relate to intracranial bleeding risk (Pantoni et al., 2014; Charidimou et al., 2016). Several studies showed that cSVD markers including cerebral microbleeds (CMBs) and white matter hyperintensities (WMH) were predictors for any ICH after IVT (Prats-Sánchez et al., 2016; Charidimou et al., 2017; Capuana et al., 2020). However, a retrospective study of 434 patients with ischemic stroke failed to demonstrate the association of white matter disease with symptomatic ICH (sICH) after IVT (Capuana et al., 2020). Recently, the role of enlarged perivascular spaces at basal ganglia (BG-EPVS) or centrum semiovale (CSO-EPVS) in ICH risk has been explored (Arba et al., 2017; Duperron et al., 2019). To date, the effect of total cSVD burden on the risk of ICH after IVT in patients with acute ischemic stroke undergoing rt-PA thrombolysis remains unclear. Therefore, we aimed to investigate the relationship between total cSVD score and the risk of ICH after IVT in a retrospective observational study.

## Materials and Methods

### Study Design, Participants, and Clinical Data

This study is a retrospective observational study performed at two teaching hospitals (Union Hospital and Longyan First Hospital) of Fujian Medical University. We included the adult patients with confirmed acute ischemic stroke who were consecutively admitted between 1 January 2013 and 31 December 2020 based on the following criteria: (1) age > 18 years; (2) had no contraindication to IVT; and (3) receiving IVT using rt-PA within 4.5 h after the onset of symptoms. Patients were excluded if they met the following exclusion criteria: (1) did not undergo brain MRI detection 48 h within symptom onset; (2) insufficient MRI data due to poor image quality; and (3) missing data regarding baseline stroke severity assessed using the National Institutes of Health Stroke Scale (NIHSS) (Kwah and Diong, 2014). Two authors blindly extracted the epidemiological, demographic, clinical, and laboratory data on admission and treatment data using a standardized data collection form. Hypertension was defined as documented systolic blood pressure ≥ 140 mmHg and/or diastolic blood pressure ≥ 90 mmHg, or by patients’ self-reported diagnosis of hypertension and/or by the treatment of antihypertensives (Sacco et al., 2011; Du et al., 2020b). Diabetes was defined as the current use of antidiabetic agents, having a fasting glucose level ≥ 7 mmol/L, having a non-fasting glucose level ≥ 11.1 mmol/L, or having a history of diabetes. Patients reporting current use of any type of tobacco during the past year were *defined* as *current* smokers (Sacco et al., 2011). Those who consumed any dose of alcohol at least one time per week during the past year were defined as *regular* alcohol users (Du et al., 2020b). We classified patients as having atrial fibrillation (AF) if they had been diagnosed with AF by electrocardiogram at admission or during the hospital stay *or had a preadmission history* (Emdin et al., 2017; Du et al., 2020b). We assessed medical history of TIA, ischemic stroke, chronic heart failure, and ischemic heart disease by preadmission history and medical records. We obtained the information of prior use of medications as a chronic intake until the admission date.

### Intravenous Thrombolysis Process

Eligible patients were treated with IVT using rt-PA (Boehringer Ingelheim Pharma GmbH) with and without bridging endovascular therapy based on the China stroke guideline (Chinese Society of Neurology and Chinese Stroke Society, 2018) and the best practice of the treating clinicians. The current China stroke guideline recommends rt-PA thrombolysis with a dose of 0.9 mg/kg. However, in clinical practice, some clinicians prefer to give a dose of 0.6 mg/kg to those with a higher ICH risk.

### Outcomes

Patients routinely underwent head computed tomography (CT) 24 h within IVT and 7 days after IVT to assess the occurrence of intracranial hemorrhage (ICH). Patients routinely underwent MRI within 48 h after onset to assess the location and size of infarction. Our main outcomes include any ICH, sICH, and remote parenchymal hemorrhages (rPH) after IVT, determined by a trained radiologist (JW) and a clinician (QY) using structured interviews blinded to baseline characteristics. sICH after IVT was defined as any subsequent visible hemorrhage associated with any neurological deterioration within 36 h of IVT, based on the National Institute of Neurological Disorders and Stroke (NINDS) criteria (Nisar et al., 2019). rPH was defined as single or multiple ICHs without visible ischemic damage detected by cranial CT, remote from the infarction area (Prats-Sánchez et al., 2016). Any disagreement was settled by referring to one senior clinician (NL) and radiologist (GC).

### Magnetic Resonance-Visible Cerebral Small Vessel Disease Assessment

All participants underwent brain MRI using 1.5 Tesla MRI scanners (Symphony Vision, Siemens Healthcare, Germany; GE Healthcare, United States) within 48 h after symptom onset. Slice thickness was 5 mm with a 1.5-mm gap between slices. Standardized parameters for the MRI sequences are as follows: T1WI sequence: repetition time (TR), 1,990 ms; echo time (TE), 8.7 ms, field of view (FOV), 230 × 217 mm^2^; T2WI sequence: TR, 4,700 ms; TE, 109 ms; FOV, 230 × 217 mm^2^; fluid-attenuated inversion recovery (FLAIR) sequence: TR, 9,000 ms; TE, 95 ms; FOV, 230 × 217 mm^2^; diffusion-weighted imaging (DWI) sequence: TR, 3,570 ms; TE, 67 ms; FOV, 235 × 235 mm^2^. We determined well-established baseline imaging cSVD markers according to the previously published method (Wardlaw et al., 2013; Capuana et al., 2020; Du et al., 2020a). Lacunes of presumed vascular origin were round or oval, located in the subcortex, similar to the signal of cerebrospinal fluid between 3 and 15 mm in diameter at fluid-attenuated inversion recovery (T2-FLAIR) and usually with low intensity of central cerebrospinal fluid and high-intensity margin around it (Wardlaw et al., 2013). CMBs were a round or oval low-signal intensity focus of cerebral parenchyma on T2-weighted gradient-recalled echo (T2*-GRE) or susceptibility-weighted imaging (SWI) with a diameter of 2–10 mm (Wardlaw et al., 2013; Capuana et al., 2020). Enlarged perivascular spaces at basal ganglia (BG-EPVS) and centrum semiovale (CSO-EPVS) were defined on T2-weighted using a validated five-point ordinal scale as follows: 0 = no EPVS, 1 = 1–10 EPVS, 2 = 11–20 EPVS, 3 = 21–40 EPVS, and 4 = > 40 EPVS (Wardlaw et al., 2013; Du et al., 2020a). We graded WMH in periventricular and deep white matter on T2-FLAIR sequences according to the Fazekas criteria (Staals et al., 2014). Moderate to severe WMH was defined as a Fazekas score *of* 3 in periventricular white matter and/or *2–3* in deep white matter. Based on a well-validated method, we calculated a total cSVD score (0–4 points) to reflect the total cSVD burden, in which one score was defined as any of the following: ≥ 1 lacune; ≥ 1 CMB; ≥ 11 BG-EPVS; moderate to severe WMH (Staals et al., 2014; Du et al., 2021).

### Statistical Analysis

Categorical variables are summarized as absolute counts with percentages. Continuous variables were expressed as means (*SD*) if normally distributed or medians (interquartile range, IQR) if not normally distributed. We used the coefficient kappa test to evaluate the inter-rater and intra-rater reliabilities regarding MRI-visible cSVD assessment. The *t*-test or Wilcoxon test, where appropriate, were used to compare the difference in continuous variables, and the chi-square test or Fisher’s exact test were used to compare the differences in categorical variables. We modeled the cSVD score on an ordinal scale continuously. Similar to several previous studies (Yilmaz et al., 2018; de Heus et al., 2020), cSVD scores of 3 and 4 were combined due to a very small number of cases (*n* = 17 and *n* = 3, respectively). Multivariate logistic regression models were used to evaluate the association between the risk of any ICH after IVT and total cSVD score (0 vs. 1, 2, 3, 4). Other variables in the multivariable logistic regression analysis were first screened using univariable analysis with *P* < 0.2 and then using a penalized regression method known as the least absolute shrinkage and selection operator (LASSO) (Tibshirani, 1996). An optimal value of the penalization parameter (lambda) for this model was obtained by 1-SE criteria using 10-fold cross-validation (Tian et al., 2020). We additionally performed an *E*-value analysis to fully explain away a specific exposure-outcome association. The *E*-value is defined as the minimum strength of association between an unmeasured confounder and an exposure/outcome to explain the observed association as non-causal (VanderWeele and Ding, 2017). We calculated the *E*-value using an online calculator.^1^ The predictive performance of a combination of NIHSS and cSVD score for any ICH after IVT was evaluated by calculating the area under the receiver operating characteristic curve (AUROC) and its 95% confidence intervals (95% CI). All statistical analyses were performed using SPSS for Windows (version 25.0, IBM), STATA (version 15.0, Stata Corp., LP), and R version 4.5 (R Foundation, Vienna, Austria) with the ‘‘glmnet’’ package.^2^ A *P*-value < 0.05 was considered statistically significant.

## Results

A total of 785 patients with acute ischemic stroke underwent IVT between 1 January and 31 December 2020. After excluding 514 patients [63.5% men, median age 69 (IQR 60–76)] who did not have baseline MRI data or had insufficient MRI data, we included 271 eligible patients [67 years (IQR 59–75); 67.1% men] in the final analysis.

Supplementary Table 1 summarizes the prevalence of individual SVD markers. Regarding the total cSVD score, 81 (29.9%) patients had a score of 0, 99 (36.5%) patients had a score of 1, 71 (26.2%) patients had a score of 2, and 20 patients (7.4%) had a score of 3–4. Supplementary Table 2 shows that the intra-rater and inter-rater reliability for individual cSVD markers were satisfactory.

Notably, 55 (20.3%) out of 271 patients experienced any ICH after IVT, 16 (5.9%) patients experienced a sICH, and 5 (1.9%) patients had rPH. Table 1 summarizes the baseline clinical and imaging characteristics among patients with and without any ICH after IVT. Patients with any ICH after IVT were older [70 (63–76) vs. 66 (58–740), *P* = 0.017], more likely to have atrial fibrillation [29 (52.7%) vs. 42 (19.4%), *P* < 0.001], a higher NIHSS score [11 (7–14) vs. 4 (2–8), *P* < 0.001], and undergo endovascular therapy [16 (29.0%) vs. 8 (3.7%), *P* < 0.001]. Regarding cSVD markers, patients with any ICH after IVT were more likely to have ≥ 1 CMB presence [35 (63.6%) vs. 60 (27.7%)], ≥ 11 BG-EPVS presence [38 (69.0%) vs. 108 (50.0%)], moderate to severe WMH presence [10 (18.1%) vs. 17 (7.8%)], and a higher cSVD score (*P* < 0.001). We found similar ≥ 1 lacune presence between patients with and without any ICH after IVT [8 (14.5%) vs. 31 (14.3%)]. Table 2 shows that patients with rPH were more likely to have lacunes [3 (60.0%) vs. 36 (13.5%)], moderate to severe WMH [2 (40.0%) vs. 25 (9.3%)], and a higher cSVD score. There were no significant differences in individual SVD markers or total cSVD score among those with and without sICH (Table 3).

**TABLE 1:** Baseline clinical and radiological characteristics for patients with and without any ICH after IVT.

	Any ICH (*n* = 55)	No any ICH (*n* = 216)	*P*-value
Age, yr (IQR)	70 (63–76)	66 (58–74)	0.017
Male, *n* (%)	35 (63.6%)	147 (68.0%)	0.534
*Current* smoker, *n* (%)	18 (32.7%)	96 (44.4%)	0.118
*Regular* alcohol user, *n* (%)	20 (36.3%)	75 (34.7%)	0.820
Previous TIA, *n* (%)	1 (1.8%)	2 (0.9%)	0.580
Previous ischemic stroke, *n* (%)	5 (9.1%)	26 (12.0%)	0.541
Hypertension, *n* (%)	41 (74.5%)	139 (64.3%)	0.155
Diabetes mellitus, *n* (%)	9 (16.4%)	48 (22.2%)	0.343
Chronic heart failure, *n* (%)	13 (23.6%)	44 (20.3%)	0.596
Ischemic heart disease, *n* (%)	10 (18.2%)	21 (9.7%)	0.083
Atrial fibrillation, *n* (%)	29 (52.7%)	42 (19.4%)	<0.001
Prior use of antiplatelet agents, *n* (%)	2 (3.6%)	23 (10.6%)	0.127
Prior use of antihypertensive agents, *n* (%)	25 (45.5%)	86 (39.8%)	0.448
Prior use of statins *n* (%)	0	11 (5.0%)	-
Prior use of hypoglycemic agents, *n* (%)	5 (9.1%)	22 (10.1%)	0.809
Prior use of anticoagulants, *n* (%)	0	7 (3.2%)	-
**DWI pattern 1, *n* (%)**			
Single Scattered Multiple territory	21 (38.1%) 23 (41.8%) 11 (20.0%)	139 (64.3%) 44 (20.3%) 33 (15.2%)	<0.001
**DWI pattern 2, *n* (%)**			
Anterior circulation Posterior circulation Mixed	45 (81.8%) 4 (7.2%) 6 (10.9%)	150 (69.4%) 44 (20.3%) 22 (10.1%)	0.094
**ASCO stroke classification, *n* (%)**			
Small vessel disease Atherothrombosis Cardioembolism Other causes	4 (7.3%) 21 (38.1%) 22 (40.0%) 8 (14.5%)	35 (16.2%) 71 (32.8%) 49 (22.7%) 61 (28.2%)	0.016
Onset to treatment time^||^, min (IQR)	175 (120–228)	180 (120–230)	0.517
Endovascular therapy, *n* (%)	16 (29.0%)	8 (3.7%)	<0.001
NIHSS score (IQR)	11 (7–14)	4 (2–8)	<0.001
Systolic blood pressure, mmHg (IQR)	155 (136–174)	152 (140–168)	0.690
Diastolic blood pressure, mmHg (IQR)	91 (82–106)	92 (82–101)	0.814
Prothrombin time*, s (IQR)	12.5 (11.8–13.0)	12.3 (11.4–13.0)	0.691
Creatinine^†^, mmol/L (IQR)	72.0 (58.5–83.0)	72.0 (60.4–84.5)	0.550
Glucose before AIS^‡^, mmol/L (IQR)	7.1 (6.3–8.4)	6.65 (5.78–8.5)	0.566
Fasting blood-glucose^§^, mmol/L (IQR)	5.6 (4.8–6.6)	5.4 (4.9–6.6)	0.931
Low density lipoprotein cholesterin^#^, mmol/L (mean ± *SD*)	2.96 ± 0.90	3.06 ± 1.02	0.522
Triglyceride^¶^, mmol/L (IQR)	1.1 (0.8–1.4)	1.2 (0.9–1.7)	0.110
Cholesterol**, mmol/L (mean ± *SD*)	4.51 ± 1.08	4.58 ± 1.16	0.664
**SVD score (0–4), *n* (%)**			
0 1 2 3–4	7 (12.7%) 15 (27.2%) 23 (41.8%) 10 (18.1%)	74 (34.2%) 84 (38.8%) 48 (22.2%) 10 (4.6%)	<0.001
≥ 1CMBs presence, *n* (%)	35 (63.6%)	60 (27.7%)	<0.001
CMBs absence, *n* (%) CMBs strictly lobar, *n* (%) CMBs strictly deep, *n* (%) CMBs mixed, *n* (%)	20 (36.3%) 17 (30.9%) 12 (21.8%) 6(10.9%)	156 (72.2%) 24 (11.1%) 26 (12.0%) 10(4.6%)	<0.001
≥ 1 Lacunes presence, *n* (%)	8 (14.5%)	31 (14.3%)	0.971
≥11BG-EPVS presence, *n* (%)	38 (69.0%)	108 (50.0%)	0.012
≥ 11 CSO-EPVS presence, *n* (%)	47 (85.4%)	176 (81.4%)	0.492
Moderate to severe WMH presence, *n* (%)	10 (18.1%)	17 (7.8%)	0.027

*IQR, interquartile range; TIA, transient ischemic attack; DWI, diffusion-weighted imaging; ASCO, atherosclerosis-small vessel disease-cardiac source-other cause; SVD, small vessel disease; BG-EPVS, basal ganglia perivascular spaces; CSO-EPVS, centrum semiovale perivascular spaces; CMBs, cerebral microbleeds; WMH, white matter hyperintensities.*

*Data of prothrombin time were available in 55 patients with any ICH and 211 patients without any ICH.

*^†^Data of creatinine were available in 53 patients with any ICH and 209 patients without any ICH.*

*^‡^Data of glucose before AIS were available in 51 patients with any ICH and 194 patients without any ICH.*

*^§^ Data of fasting blood-glucose were available in 51 patients with any ICH and 192 patients without any ICH.*

*^||^Data of onset to treatment time were available in 45 patients with any ICH and 175 patients without any ICH.*

*^#^Data of low-density lipoprotein cholesterin were available in 53 patients with any ICH and 213 patients without any ICH.*

*^¶^ Data of triglyceride were available in 53 patients with any ICH and 213 patients without any ICH.*

**Data of cholesterol were available in 53 patients with any ICH and 213 patients without any ICH.

**TABLE 2:** Baseline clinical and radiological characteristics for patients with and without rPH after IVT.

	rPH (*n* = 5)	No rPH (*n* = 266)
Age, yr (IQR)	70 (57–85)	67 (59–75)
Male, *n* (%)	3 (60.0%)	179 (67.2%)
*Current* smoker, *n* (%)	2 (40.0%)	112 (42.1%)
*Regular* alcohol user, *n* (%)	2 (40.0%)	93 (34.9%)
Previous TIA, *n* (%)	0	3 (1.1%)
Previous ischemic stroke, *n* (%)	0	31 (11.6%)
Hypertension, *n* (%)	5 (100%)	175 (65.7%)
Diabetes mellitus, *n* (%)	0	57 (21.4%)
Chronic heart failure, *n* (%)	2 (40.0%)	55 (20.6%)
Ischemic heart disease, *n* (%)	2 (40.0%)	29 (10.9%)
Atrial fibrillation, *n* (%)	1 (20.0%)	70 (26.3%)
Prior use of antiplatelet agents, *n* (%)	0	25 (9.3%)
Prior use of antihypertensive agents, *n* (%)	2 (40.0%)	109 (40.9%)
Prior use of statins *n* (%)	0	11 (4.1%)
Prior use of hypoglycemic agents, *n* (%)	0	27 (10.1%)
Prior use of anticoagulants, *n* (%)	0	7 (2.6%)
**DWI pattern 1, *n* (%)**		
Single Scattered Multiple territory	3 (60.0%) 1 (20.0%) 1 (20.0%)	157 (59.0%) 66 (24.8%) 43 (16.1%)
**DWI pattern 2, *n* (%)**		
Anterior circulation Posterior circulation Mixed	5 (100%) 0 0	190 (71.4%) 48 (18.0%) 28 (10.5%)
**ASCO stroke classification, *n* (%)**		
Small vessel disease Atherothrombosis Cardioembolism Other causes	1 (20.0%) 1 (20.0%) 1 (20.0%) 2 (40.0%)	38 (14.2%) 91 (34.2%) 70 (26.3%) 67 (25.1%)
Onset to treatment time^||^, min (IQR)	126 (75–213)	180 (121–230)
Endovascular treatment, *n* (%)	1 (20.0%)	23 (8.6%)
NIHSS score (IQR)	6 (4–12)	5 (3–10)
Systolic blood pressure, mmHg (IQR)	150 (120–185)	153 (140–168)
Diastolic blood pressure, mmHg (IQR)	111 (77–119)	92 (82–101)
Prothrombin time*, s (IQR)	12.7 (6.4–13.8)	12.3 (11.5–13.0)
Creatinine^†^, mmol/L (IQR)	72.0 (69.3–79.3)	72.0 (58.9–84.0)
Glucose before AIS^‡^, mmol/L (IQR)	6.7 (5.6–9.5)	6.7 (5.9–8.4)
Fasting blood-glucose^§^, mmol/L (IQR)	5.2 (4.5–6.6)	5.5 (4.9–6.6)
Low density lipoprotein cholesterin^#^, (mmol/L), mean ± *SD*	2.68 ± 1.31	3.04 ± 1.00
Triglyceride^¶^, mmol/L (IQR)	1.2 (0.7–2.9)	1.2 (0.9–1.7)
Cholesterol**, mmol/L, mean ± *SD*	4.37 ± 2.10	4.57 ± 1.12
SVD score (0–4), *n* (%)		
0 1 2 3–4	0 0 3 (60.0%) 2 (40.0%)	81 (30.4%) 99 (37.2%) 68 (25.5%) 18 (6.7%)
≥ 1 CMBs, *n* (%)	3 (60.0%)	92 (34.5%)
CMBs absence, *n* (%) CMBs strictly lobar, *n* (%) CMBs strictly deep, *n* (%) CMBs mixed, *n* (%)	2 (40.0%) 2 (40.0%) 0 1 (20.0%)	174 (65.4%) 39 (14.6%) 38 (14.2%) 15 (5.6%)
≥ 1 Lacunes, *n* (%)	3 (60.0%)	36 (13.5%)
≥11BG-EPVS presence, *n* (%)	4 (80.0%)	142 (53.3%)
≥ 11 CSO-EPVS presence, *n* (%)	4 (80.0%)	219 (82.3%)
Moderate to severe WMH presence, *n* (%)	2 (40.0%)	25 (9.3%)

*IQR, interquartile range; TIA, transient ischemic attack; DWI, diffusion-weighted imaging; ASCO, atherosclerosis-small vessel disease-cardiac source-other cause; SVD, small vessel disease; BG-EPVS, basal ganglia perivascular spaces; CSO-EPVS, centrum semiovale perivascular spaces; CMBs, cerebral microbleeds; WMH, white matter hyperintensities.*

*Data of prothrombin time were available in 5 patients with rPH and 261 patients without rPH.

*^†^Data of creatinine were available in 5 patients with rPH and 257 patients without rPH.*

*^‡^Data of glucose before AIS were available in 5 patients with rPH and 240 patients without rPH.*

*^§^ Data of fasting blood-glucose were available in 5 patients with rPH and 238 patients without rPH.*

*^||^Data of onset to treatment time were available in 4 patients with rPH and 216 patients without rPH.*

*^#^Data of low-density lipoprotein cholesterin were available in 5 patients with rPH and 261 patients without rPH.*

*^¶^ Data of triglyceride were available in 5 patients with rPH and 261 patients without rPH.*

**Data of cholesterol were available in 5 patients with rPH and 261 patients without rPH.

**TABLE 3:** Baseline clinical and radiological characteristics for patients with and without sICH.

	sICH (*n* = 16)	No sICH (*n* = 255)	*P-*value
Age, yr (IQR)	70 (64–76)	67 (58–75)	0.182
Male, *n* (%)	10 (62.5%)	172 (67.5%)	0.683
*Current* smoker, *n* (%)	3 (18.7%)	111 (43.5%)	0.051
*Regular* alcohol user, *n* (%)	5 (31.2%)	90 (35.2%)	0.742
Previous TIA, *n* (%)	1 (6.2%)	2 (0.8%)	0.426
Previous ischemic stroke, *n* (%)	2 (12.5%)	29 (11.4%)	> 0.999
Hypertension, *n* (%)	13 (81.3%)	167 (65.5%)	0.195
Diabetes mellitus, *n* (%)	1 (6.2%)	56 (22.0%)	0.238
Chronic heart failure, *n* (%)	3 (18.8%)	54 (21.2%)	> 0.999
Ischemic heart disease, *n* (%)	2 (12.5%)	29 (11.4%)	> 0.999
Atrial fibrillation, *n* (%)	10 (62.5%)	61 (23.9%)	0.002
Prior use of antiplatelet agents, *n* (%)	2 (12.5%)	23 (9.0%)	0.983
Prior use of antihypertensive agents, *n* (%)	7 (43.8%)	104 (40.8%)	0.815
Prior use of statins *n* (%)	0	11 (4.3%)	0.845
Prior use of hypoglycemic agents, *n* (%)	0	27 (10.6%)	0.346
Prior use of anticoagulants, *n* (%)	0	7 (2.7%)	> 0.999
**DWI pattern 1, *n* (%)**			
Single Scattered Multiple territory	4 (25.0%) 7 (43.8%) 5 (31.3%)	156 (61.2%) 60 (23.5%) 39 (15.3%)	0.017
**DWI pattern 2, *n* (%)**			
Anterior circulation Posterior circulation Mixed	11 (68.8%) 3 (18.8%) 2 (12.5%)	185 (72.5%) 45 (17.6%) 26 (10.2%)	0.948
**ASCO stroke classification, *n* (%)**			
Small vessel disease Atherothrombosis Cardioembolism Other causes	1 (6.3%) 6 (37.5%) 6 (37.5%) 3 (18.8%)	38 (14.9%) 86 (33.7%) 65 (25.5%) 66 (25.9%)	0.550
Onset to treatment time^||^, min (IQR)	190 (131–254)	180 (120–230)	0.575
Endovascular treatment, *n* (%)	6 (37.5%)	18 (7.1%)	< 0.001
NIHSS score (IQR)	12 (6–16)	5 (3–10)	0.001
Systolic blood pressure, mmHg (IQR)	154 (141–175)	152 (139–168)	0.782
Diastolic blood pressure, mmHg (IQR)	89 (77–105)	92 (82–101)	0.451
Prothrombin time*, s (IQR)	12.8 (12.3–13.6)	12. 3 (11.5–13.0)	0.028
Creatinine^†^, mmol/L (IQR)	71.0 (57.7–83.7)	72.0 (59.9–84.0)	0.920
Glucose before AIS^‡^, mmol/L (IQR)	6.5 (5.7–8.6)	6.7 (5.9–8.4)	0.585
Fasting blood-glucose^§^, mmol/L (IQR)	6.2 (4.9–6.7)	5.5 (4.9–6.6)	0.497
Low density lipoprotein cholesterin^#^, mmol/L, mean ± *SD*	3.20 ± 0.83	3.03 ± 1.01	0.540
Triglyceride^¶^, mmol/L (IQR)	1.2 (0.8–1.5)	1.2 (0.9–1.7)	0.557
Cholesterol**, mmol/L, mean ± SD	4.84 ± 1.14	4.55 ± 1.14	0.345
**SVD score (0–4), *n* (%)**			
0 1 2 3–4	5 (31.3%) 3 (18.8%) 5 (31.3%) 3 (18.8%)	76 (29.8%) 96 (37.6%) 66 (25.9%) 17 (6.7%)	0.322
≥ 1 CMBs, *n* (%)	7 (43.8%)	88 (34.5%)	0.452
CMBs absence, *n* (%) CMBs strictly lobar, *n* (%) CMBs strictly deep, *n* (%) CMBs mixed, *n* (%)	9 (56.3%) 4 (25.0%) 3 (18.8%) 0	167 (65.5%) 37 (14.5%) 35 (13.7%) 16 (6.3%)	0.344
≥1 Lacunes, *n* (%)	4 (25.0%)	35 (13.7%)	0.379
≥11BG-EPVS presence, *n* (%)	10 (62.5%)	136 (53.3%)	0.476
≥11 CSO-EPVS presence, *n* (%)	16 (100%)	207 (81.2%)	0.115
Moderate to severe WMH presence, *n* (%)	1 (6.3%)	26 (10.2%)	0.935

*IQR, interquartile range; TIA, transient ischemic attack; DWI, diffusion-weighted imaging; ASCO, atherosclerosis-small vessel disease-cardiac source-other cause; SVD, small vessel disease; BG-EPVS, basal ganglia perivascular spaces; CSO-EPVS, centrum semiovale perivascular spaces; CMBs, cerebral microbleeds; WMH, white matter hyperintensities.*

*Data of prothrombin time were available in 16 patients with sICH and 250 patients without sICH.

*^†^Data of creatinine were available in 16 patients with sICH and 246 patients without sICH.*

*^‡^Data of glucose before AIS were available in 16 patients with sICH and 229 patients without sICH.*

*^§^ Data of fasting blood-glucose were available in 16 patients with sICH and 227 patients without sICH.*

*^||^Data of onset to treatment time were available in 14 patients with sICH and 206 patients without sICH.*

*^#^Data of low-density lipoprotein cholesterin was available in 15 patients with sICH and 251 patients without sICH.*

*^¶^ Data of triglyceride were available in 15 patients with sICH and 251 patients without sICH.*

**Data of cholesterol were available in 15 patients with sICH and 251 patients without sICH.

**FIGURE 1:**
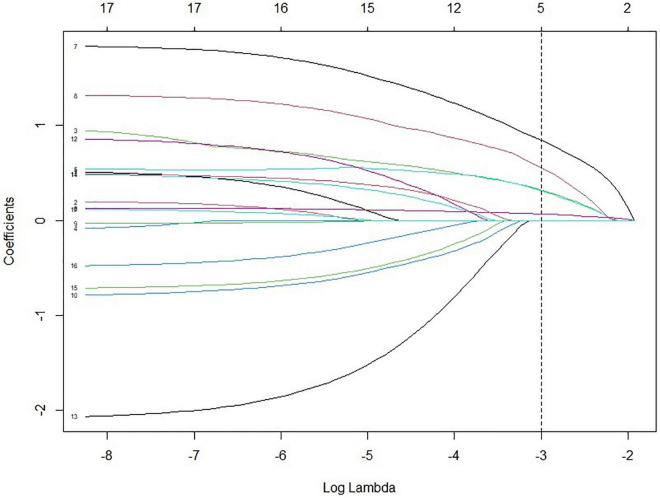
Variables selected by LASSO from 17 characteristics with *P* < 0.2 from the univariable analysis. (1) Perivascular spaces at basal ganglia; (2) Moderate to severe white matter hyperintensities; (3) ≥ 1 CMBs; (4) CMB location (strict lobe, strict deep, and mixed); (5) cSVD score (0 vs. 1, 2, 3, 4); (6) NIHSS; (7) endovascular therapy; (8) atrial fibrillation; (9) age (per year increase); (10) current smoker; (11) hypertension; (12) ischemic heart disease; (13) prior use of antiplatelet treatment; (14) DWI mode 1 (single, scattered, and multiple); (15) DWI mode 2 (anterior circulation stroke, posterior circulation stroke, and mixed); (16) triglyceride; (17) ASCO stroke subtype. LASSO, least absolute shrinkage and selection operator; ASCO, atherothrombosis, small vessel disease, cardioembolism, other; NIHSS, National Institutes of Health Stroke Scale.

**FIGURE 2:**
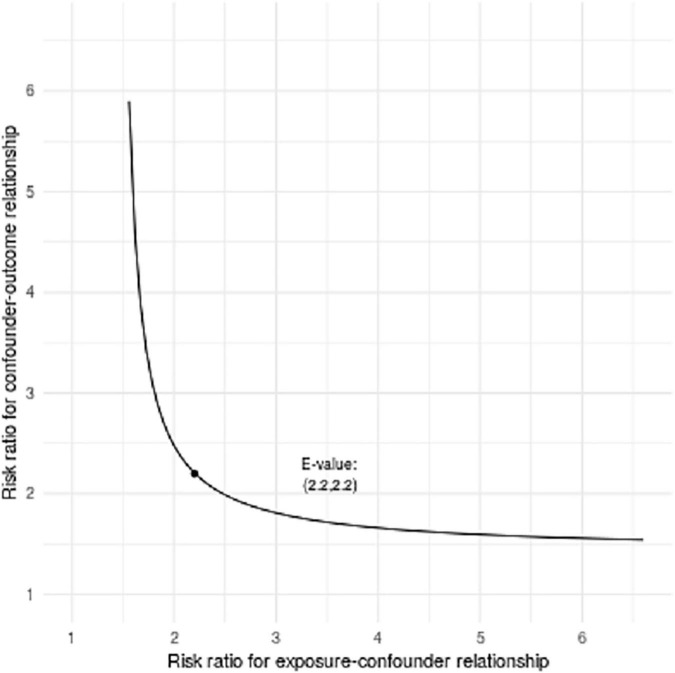
Bias plots assessed by *E*-value analysis (outcome is any ICH). Bias plots represent varying levels of unobserved confounder/exposure association and unobserved confounder/outcome association necessary to render observed exposure/outcome association null for total cSVD score per point increase.

The LASSO model filtered out four variables (i.e., NIHSS, endovascular therapy, AF, and total cSVD score) from 17 risk characteristic individual factors for any ICH in the final multivariable regression model. Figure 1 shows how the regression coefficients change as a function of the penalization parameter [lambda (λ)]. On the far left, we saw the coefficients from standard logistic regression. As we go from left to right, the coefficients are “shrunk” toward zero. The coefficient estimated from the optimal model is indicated by the dashed line, which shows that only NIHSS, endovascular therapy, AF, and the total cSVD score were kept in the model. Multivariable logistic regression analysis showed that the total cSVD score (per unit increase, OR 2.03 95% 1.22–3.41, *P* = 0.007) was significantly associated with a higher risk of any ICH after IVT. The risk of any ICH increased with increasing cSVD score (cSVD score 1, OR 1.29, 95% CI 0.44–3.94, cSVD score 2, OR 4.90, 95% CI 1.81–14.81, cSVD 3–4, OR 12.40, 95% CI 3.40–48.77, compared with a score of 0, *P* < 0.001, AUC 0.698, 95% CI 0.620–0.777, Table 4). The very small number of sICH (*n* = 16) and rPH events (*n* = 5) did not allow us to perform the logistic regression analysis using the LASSO approach. Sensitivity analyses using the *E*-value revealed that it would need moderately robust unobserved confounding to render the exposure-outcome (cSVD-any ICH) association null (Figure 2).

**TABLE 4:** Association of cSVD score and any ICH risk after IVT using LASSO multivariate logistic regression analysis.

	Multivariable logistic regression analysis	
Variables	OR (95% CI)	*P*-value
cSVD score (0–4) 0 1 2 3–4	Ref 1.29 [0.44–3.94] 4.90 [1.81–14.81] 12.40 [3.40–48.78]	< 0.001*
Continuous cSVD sum score	2.03 [1.22–3.41]	0.007*

*cSVD, cerebral small vessel disease; ICH, intracerebral hemorrhage; IVT, intravenous thrombolysis; LASSO, least absolute shrinkage and selection operator.*

*Adjusted for NIHSS: atrial fibrillation and endovascular therapy.

**FIGURE 3:**
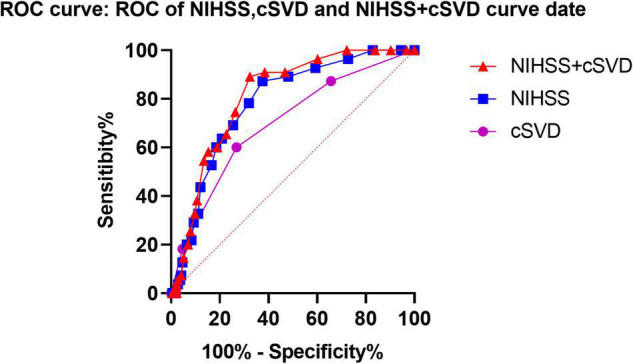
Predictive performances of NIHSS score, SVD burden and a combined NIHSS score, and SVD burden using ROC analysis. ROC, receiver operating characteristic; cSVD, cerebral small vessel disease.

Based on a previous meta-analysis and our LASSO selection results, the NIHSS score was considered a robust predictor for any ICH after IVT (Whiteley et al., 2012). Therefore, we compared the predictive performance of a combination of NIHSS and cSVD score with that of the NIHSS score alone. The results showed that combining cSVD and NIHSS score had a better predictive performance than NIHSS score alone (AUC 0.811, 95% CI 0.756–0.866 vs. 0.784, 95% CI 0.723–0.846, *P* = 0.0004, Table 5 and Figure 3).

**TABLE 5:** Predictive performance of total cSVD score adds to the NIHSS score using ROC analysis.

	AUC	95% CI	*P*-value	Youden’s index
Total SVD score	0.698	0.620–0.777	<0.001	1.5
NIHSS score	0.784	0.723–0.846	<0.001	5.5
NIHSS + SVD score	0.811	0.756–0.866	< 0.001*	7.5

*cSVD, cerebral small vessel disease; NIHSS, National Institutes of Health Stroke Scale; ROC, receiver operating characteristic curve.*

*P = 0.0004 (comparison of AUROC of NIHSS score and a combined NIHSS score and total SVD score using Z statistics).

## Discussion

This study shows that a higher SVD burden is associated with increased risk of any ICH after IVT, suggesting that the risk of any ICH in patients with acute ischemic stroke who received IVT with rt-PA might be related to SVD severity. Moreover, the cSVD score adds to the NIHSS score alone in predicting any ICH after IVT.

In our cohort, patients with any ICH were more likely to have ≥ 1 CMB presence. However, CMB presence was not screened out using the LASSO model. The limited sample size of our study does not enable us to perform the further analysis stratified by CMB burden. Our findings were supported by several studies that showed the CMB presence was not associated with an increased risk of any ICH (Kakuda et al., 2005; Kim et al., 2006; Fiehler et al., 2007) or sICH (Charidimou et al., 2017; Capuana et al., 2020) after IVT. Current stroke guidelines do not recommend IVT in patients with > 10 CMBs at baseline (Chinese Society of Neurology and Chinese Stroke Society, 2018; Powers et al., 2018; Berge et al., 2021). A recent study using a multistep algorithm populated with data showed that in patients with > 10 CMBs, IVT is associated with higher mortality and net harm defined by age, stroke symptom severity, and treatment delay (Schlemm et al., 2020). To date, there are no studies reporting directly on the risk of sICH in patients without IVT stratified by CMB burden. Future prospective studies are needed to define the CMB burden threshold in individual risk stratification predicting the bleeding risk after IVT and functional outcome.

Evaluating the total cSVD burden may allow more accurate estimates of size effects on ICH risk after IVT than individual cSVD markers. In our cohort, the total SVD score showed a dose-effect response, reinforcing the validity of the findings. Similarly, a small sample-sized retrospective study showed that severe cSVD burden (cSVD score of 3–4) was associated with an increased risk of hemorrhagic transformation after IVT (OR = 8.429, 95% CI 1.643–43.227, *P* = 0.011) (Liu et al., 2019). Our study adds to the aforementioned study by investigating the association between total cSVD score (0 vs. 1, 2, 3, 4) and the bleeding risk after IVT, using variables that were selected by LASSO. Moreover, our data supported the possible relationship between the risk of rPH and the total cSVD score. However, the small number of rPH events did not enable us to perform the regression analysis. A previous single-center observational study showed that patients with stroke with r-PH after IVT were more likely to have strictly lobar CMBs, suggesting amyloid angiopathy (Drelon et al., 2020). Since the occurrence of r-PH may be related to worse outcomes, the association between r-PH and total cSVD score needs further investigation. Possible mechanisms that account for the role of cSVD in ICH after IVT include endothelial dysfunction, arterial stiffening (arterial stiffening might increase ICH risk), and increased blood-brain barrier permeability (Campbell and Khatri, 2020).

Consistent with a previous meta-analysis (Whiteley et al., 2012), our data support that the NIHSS score is a robust predictor for ICH risk after IVT. The larger AUC of a combined NIHSS and cSVD score than an NIHSS score alone suggests that adding cSVD as neuroimaging biomarkers to a widely validated factor (NIHSS) might improve specificity and sensitivity in predicting ICH after IVT. Since most of our cohort had mild to moderate acute ischemic stroke [median NIHSS score 5 (IQR 3–10)], our results might not be generalized to the patients with severe stroke. Future large-scale studies are required to refine and validate robust risk prediction tools.

There are some limitations of this study. First, this is a retrospective study with a limited sample size. Second, excluding patients who did not undergo MRI within 48 h after onset or insufficient MR image might introduce selection bias since most severe patients were not included. Third, we did not analyze the functional outcome data after discharge in this study; a longer follow-up period data will better determine the significance of our findings. Fourth, very few patients underwent MR before IVT; therefore, we could not exclude the new cSVD markers (particularly new CMBs) after IVT (Braemswig et al., 2019). However, the cSVD findings did not influence the IVT decisions. Fifth, due to the small number of outcome events, we did not assess the predicting performance of cSVD for sICH and rPH, which is the most clinically relevant. Finally, due to the data unavailability, we were unable to calculate the estimated effect size adjusted for some unmeasured confounders. However, we used the *E*-value sensitivity analysis to quantify the potential implications of unmeasured confounders and found that it was unlikely to explain the entire outcome.

## Conclusion

This study shows that the total cSVD score is associated with a higher risk of any ICH after IVT. However, our study was not powered to assess the association between cSVD score and sICH risk. Further studies are warranted to determine whether rt-PA can be safely initiated in patients with acute ischemic stroke who have coexistent cSVD.

## Data Availability Statement

The original contributions presented in the study are included in the article/Supplementary Material, further inquiries can be directed to the corresponding author/s.

## Ethics Statement

The studies involving human participants were reviewed and approved by the Fujian Medical University Union Hospital Ethics Committees (2019KY076). Written informed consent for participation was not required for this study in accordance with the national legislation and the institutional requirements.

## Author Contributions

HD and NL: concept, design, and full access to all of the data in this study and take responsibility for the integrity of the data and the accuracy of the data analysis. HD, SW, HhL, and NL: acquisition, analysis, and interpretation of data. HD, SW, and HfL: drafting of the manuscript. GA, DW, HfL, YC, DC, QY, SF, RC, YZ, JW, GC, and JC: critical revision of the manuscript for important intellectual content. HD, HhL, and GA: statistical analysis. All authors contributed to the article and approved the submitted version.

## Conflict of Interest

The authors declare that the research was conducted in the absence of any commercial or financial relationships that could be construed as a potential conflict of interest.

## Publisher’s Note

All claims expressed in this article are solely those of the authors and do not necessarily represent those of their affiliated organizations, or those of the publisher, the editors and the reviewers. Any product that may be evaluated in this article, or claim that may be made by its manufacturer, is not guaranteed or endorsed by the publisher.

## Footnotes

1
https://mmathur.shinyapps.io/evalue/

2
http://www.Rproject.org
