# Supplementary material for: Total Cerebral Small Vessel Disease Score and Cerebral Bleeding Risk in Patients With Acute Stroke Treated With Intravenous Thrombolysis
Source: Front Aging Neurosci. 2022 Apr 11;14:790262. doi: 10.3389/fnagi.2022.790262 (PMC9037754; doi:10.3389/fnagi.2022.790262)
Supplement: Supplementary file 1 [file Table_1.DOCX]

Supplementary Material

**Supplemental Table 1.** **The prevalence of individual cSVD markers and cSVD score**

|  | n = 271 | |
| --- | --- | --- |
| ≥1 CMBs presence, n (%) | | 95 (35.1%) |
| CMBs strictly lobar | | 41 (15.1%) |
| CMBs strictly deep | | 38 (14.0%) |
| CMBs mixed | | 16 (5.9%) |
| moderate to severe WMH, n (%) | | 27 (10.0%) |
| ≥11BG-EPVS presence, n (%) | | 146 (53.9%) |
| ≥11 CSO-EPVS presence, n (%) | | 223 (82.3%) |
| ≥1 lacunes presence, n (%) | | 39 (14.4%) |
| cSVD score(0-4), n (%) | |  |
| 0 | | 81 (29.9%) |
| 1 | | 99 (36.5%) |
| 2 | | 71 (26.2%) |
| 3 to 4 | | 20 (7.4%) |

Abbreviations: cSVD = cerebral small vessel disease; CMBs = cerebral microbleeds; WMH = white matter hyperintensities; BGPVS = basal ganglia perivascular spaces; CSOPVS = centrum semiovale perivascular spaces;

**Supplemental Table 2.** **The intra-rater and inter-rater reliability for individual cSVD markers**

|  | | intra-rater Coefficient    kappa(95%CI) | inter-rater Coefficient  kappa(95%CI) |
| --- | --- | --- | --- |
| presence of CMBs | 0.80 (0.59-1.01) | | 0.83 (0.65-1.01) |
| presence of BG-PVS | 0.68 (0.48-0.88) | | 0.72 (0.50-0.96) |
| presence of CSO-PVS | 0.69 (0.50-0.89) | | 0.67 (0.53-0.91) |
| presence of moderate to severe WMH | 0.84 (0.54-1.14) | | 0.80 (0.55-1.06) |
| presence of lacunes | 0.76 (0.46-1.07) | | 0.78 (0.37-1.19) |

Abbreviations: cSVD = cerebral small vessel disease; CMBs = cerebral microbleeds; WMH = white matter hyperintensities; BGPVS = basal ganglia perivascular spaces; CSOPVS = centrum semiovale perivascular spaces;
